# Acknowledgement to Reviewers of *Medicines* in 2018

**DOI:** 10.3390/medicines6010010

**Published:** 2019-01-15

**Authors:** Medicines Editorial Office

**Affiliations:** MDPI, St. Alban-Anlage 66, 4052 Basel, Switzerland

Rigorous peer-review is the corner-stone of high-quality academic publishing. The editorial team greatly appreciates the reviewers who contributed their knowledge and expertise to the journal’s editorial process over the past 12 months. In 2018, a total of 131 papers were published in the journal, with a median time to first decision of 15 days and a median time to publication of 33 days. Among the reviewers who reviewed for *Medicines* in 2018, the following two have been selected to receive the “*Medicines* 2018 Outstanding Reviewer Award” for the timeliness and quality of their review reports in 2018:

Sawynok, Jana from Dalhousie University, Canada

Tanaka, Tim Hideaki from Tsukuba University of Technology, Japan

The editors would also like to express their sincere gratitude to all the following reviewers for their cooperation and dedication in 2018:
Abejón, RicardoLiao, Jyh-feiAhmad, ZeeshanLin, RanAl-Fatimi, MohamedLitofsky, Norman ScottAlmeida, HugoLiu, YuAl-Rimawi, FuadLocatelli, MarcelloAltemimi, AmmarLorenzo Rodriguez, Jose ManuelAmarowicz, RyszardLovell, JonathanAmorós, AsunciónLuévano-Contreras, ClaudiaAntal, DianaLundstrom, Brian NilsAspatwar, AshokLuongo, LivioAssenza, GiovanniMabou Tagne, AlexÅstrand, AlexanderMacVicar, SonyaAy, IlknurMaietti, AnnalisaBaba, YuhManakil, JaneBaldisserotto, AnnaMandava, NandaBarreira, João C.M.Manfredini, StefanoBasak, DebasishMarchini, CristinaBasavarajappa, Balapal S.Marcovici, GenoBayin, N. SumruMarks, RayBeasley, ShannonMartinelli, PaolaBellavia, DanieleMartinez-Castelao, AlbertoBiagi, MarcoMartins, Maria RosarioBirkett, JasonMartins, NatáliaBlanton, CynthiaMazzoni, LucaBo’, Cristian DelMcConnell, TraceyBolognini, DanieleMcGuire, TreasureBondonno, Nicola P.Michel, PiotrBonomini, FrancescaMiele, ErmannoBožović, MijatMiele, Adriana E.Bradley, Elyse CornettMikropoulos, ChristosBradshaw, PatrickMiyata, YasuyoshiBravenboer, NathalieMobbili, GiovannaBravo López, Susana BMohammed, YousufBrown, MelanieMontenegro, LuciaBubb, Kristen J.Mosawy, SaphaButler, MarkMulas, MaurizioByun, JonghoeMüller, MatthiasCabral, CéliaMunshi, Nikhil V.Campbell, Fiona M.Musch, MarkCanini, AntonellaNamasivayam, VigneshwaranCapasso, FrancescoNannoni, EleonoraCapasso, RaffaeleNavran, ArashCardoso, Susana M.Nekludov, MichaelCasalino, ElisabettaNikas, Spyros P.Caster, JosephNishi, MayumiCavarra, BereniceNitulescu, George MihaiChang, Wen-DienNolan, ClodaghChatterjee, JhinukObermüller, NicholasChen, Yi-HungOchiai, HirokoChereddy, Kiran KumarOliveira-Santos, Sonia AndreiaChizzola, RemigiusOts, ThomasChobot, VladimirOzaita Mintegui, AndrésChue, Pierre S.Pacella, ElenaChurchward-Venne, Tyler A.Pangestu, MulyotoÇiçek, Serhat SezaiPapetti, AdeleCiulu, MarcoPark, Il-KwonClément-Colmou, Karen Clément-ColmouPatwardhan, Avinash R.Cohen, DanPereira, Olívia R.Collet, Jean-paulPerez-Stable, CarlosCollier, JasonPinto, MafaldaContino, MarialessandraPiper, BrianCouzinet-mossion, AureliePorter, DiannaCoyle, Patricia K.Potvin, NoahCullen, CarliePrats, Anne-CatherineCunningham-Rundles, SusannaPreethichandra, PreethiDalessandro, GiuseppePrieto-Lloret, JesusD’Argenio, ValeriaProescholdt, MartinD’Auria, EnzaPu, JingDawidowicz, A. L.Qian, LiDe Berardis, DomenicoQuandt, SaraDeng, XiaoranRaake, Philip W.J.Deriu, FrancaRagusa, AndreaDi Cesare Mannelli, LorenzoRaith, WolfgangDíaz Martínez, MargaritaRamakrishnan, SadeeshDinh, AnhRamchandani, DivyaDinu-Pîrvu, Cristina ElenaRamirez-Vick, JaimeDonadio, CarloRamsey, Vincent K.Donato, RosarioRamtekkar, UjjwalDudas, JozsefRecchia, Daniela R.Eleftheriadis, TheodorosRenner, BertoldEl-Seedi, Hesham R.Reybrouck, MarkEspuelas, SocorroRodríguez-Yoldi, María JesúsFalco, AlbertoRoe, Daniel R.Feas, XesusRossi, FilippoFine, James BurkeRuiz-Aceituno, LauraFonteh, AlfredSak, Jarosław J.Franchini, CarloSamuel, TemesgenFujita, MasakiSantacroce, LuigiGao, MinSaturnino, CarmelaGasparrini, MassimilianoSaunders, ChristobelGerhold, KerstinSawada, ShojiroGestal, MonicaSawynok, JanaGhatak, ArindamScharoun Benson, SaraGhilli, MatteoSchicho, RudolfGiamperi, LauraSchmitz, John C.Giardino, AlessandroScrano, LauraGinter, Paula S.Scrima, MarioGłąbska, DominikaSeca, Ana Maria Loureiro DaGonçalves, Lidia M DSetzer, William N.Gopalakrishnan, GopanShekhtman, AlexanderGrande, FedoraShinnick, PhillipGrant, GeorgeSholkamy, EmanGrigsby, Christopher L.Sigusch, Bernd W.Gu, ZhenSilva, Luís R.Guina, JeffreySilverman, Michael JGupta, PrachiSmith, Leonard B.Güthlin, CorinaSorce, SilviaHans, GuySoto, CarmenHanski, LeenaSpengler, DietmarHaponiuk, JózefStacewicz-Sapuntzakis, MariaHayashi, YuichiroStenager, EgonHerrero, María JesúsStout, JeffHicks, AnnSugimoto, NaotoshiHo, Wing ShingSun, WujinHochheiser, HarrySun, ShiHooda, JagmohanSvendsen, Kristina BacherHorhat, Florin GeorgeSzebeni, Gábor J.Howes, Laurence GuyTakakura, NobuariHshieh, Tammy TTakaya, JunjiHu, MinTanaka, Tim HideakiImamura, TeruhikoTanaka, ToshiakiInvitto, SaraTarantino, GiovanniIsola, GaetanoTaylor, MatthewJadhav, Gopal P.Tellez, Trinidad RuízJanakiraman, HarinarayananTeschke, RolfJaradat, NidalThompson, Miles D.Jaschke, Artur CThompson, BillJayant, KumarTimmerman, KyleJohansen, Guro GravemToillon, Robert-AlainK Song, MoonTourniaire, FranckKandala, Ngianga-BakwinTrivedi, DarshanKantor, JiriTsermpini, Evangelia EiriniKarmakar, ParthaTullius Scotti, MarcusKathuria, HimanshuUmerska, AnitaKennedy, Mary-ClaireUzan, PierreKhairullina, VeronikaVafiadaki, ElizabethKim, Won HVan De Ven, RienekeKim, Ki HyunVarela-López, AlfonsoKim, Ji YeonVaughan, Christopher W.Kim, Yun-baeVázquez Tato, M. PilarKim, DaehwanVitalini, SaraKimura, Ken-ichiWakeling, SimonKöfalvi, AttilaWanchai, AusaneeKopsky, David J.Wang, Tsung-JenKoutelidakis, Antonios E.Wang, Jong-ShyanKról, EwelinaWang, YifeiKujan, OmarWang, Chin-KunKumar, SunilWilkinson, J. M.Lalas, StavrosXu, HuichunLaPaglia, DonnaXuan, Tran DangLaronga, ChristineYadav, DhananjayLatunde-Dada, Gladys OluyemisiYang, LinLauritano, DorinaYang, Wei-hsiungLeal, Ana MendesYuan, ZhihongLee, Hyeon YongZhang, QiuyangLee, Sang-HanZhang, ChiLesiuk, TeresaZhang, FanLevy, StevenZhou, QingxiangLi, ShaoZou, LiyeLi, JieZucca, Paolo

